# Supplementary material for: Evaluation of whole exome sequencing as an alternative to BeadChip and whole genome sequencing in human population genetic analysis
Source: BMC Genomics. 2018 Oct 29;19:778. doi: 10.1186/s12864-018-5168-x (PMC6206721; doi:10.1186/s12864-018-5168-x)
Supplement: Supplementary file 1 — Table S1. Number of high-quality biallelic variants in GENOME, EXOME, BEADCHIP, and HUN-EXOM datasets before and after LD pruning. Table S2. f3-statistics of Hungarian and selected South Asian populations indicate admixture which could be attributed to the Gypsies living as an ethnic minority in Hungary. Figure S1. The f4 (TSI, X; CHB; YRI) statistics estimates the East Asian and African components for each dataset. Negative values indicates East Asian gene flow, while positive values indicate dominant African genetic components. Figure S2. Calculated CV error for different admixture models (K=3 to 10). The best fitting model of admixture indicated was K=6 in the GENOME, EXOME and HUN-EXOME datasets, however, it was K=9 in case of the BEADCHIP dataset. Figure S3. Admixture analysis of 25 populations with the K=6 admixture model. The major topology and admixture components were very similar in each dataset for all analyzed populations but the minor admix components were systematically overestimated using the BEADCHIP dataset. Figure S4. Admixture analysis of 25 populations with the K=9 admixture model. Besides the minor admix components overestimation using BEADCHIP this admixture model suggested that the European super-population was split into two and the African super-population was split into three admixing components. Figure S5. PCA of the HUN-EXOME dataset confirms that genetically, modern Hungarians belong to the European super-population and within the Europeans, two genetic components exist with a North-South European gradient. Figure S6. F4(TSI, X; CHB, YRI) values of the HUN-EXOME dataset indicates that Hungarians have higher East Asian admixture component than British but lower than Finnish population. Figure S7. Admixture analysis of 25 populations using the HUN-EXOME dataset for the A) K=6 and B) K=9 admixture models. Besides the major Europian admixture component in few Hungarian individuals minor East Asian admixture components also detected. Data Sup [file 12864_2018_5168_MOESM1_ESM.pdf]

Supplementary information

Contents

Table S1 2

Table S2 3

Figure S1 4

Figure S2 5

Figure S3 6

Figure S4 7

Figure S5 8

Figure S6 9

Figure S7 10

Data 11

Table S1

Number of high-quality biallelic variants in different datasets before and after LD pruning.

dataset	biallelic variants				
	unpruned	pruned			
		sliding window		difference between pruning windows	
		50k	10000k	count	percent (%)
GENOME	483100	232194	206253	25941	11.17%
EXOME	405496	184258	149453	34805	18.89%
BEADCHIP	567187	79492	72701	6791	8.54%
HUN-EXOME	408790	186984	151259	35725	19.11%

The unpruned datasets were LD pruned by Plink with two different sliding windows (50k and 10,000k respectively), and the difference of pruned markers between the two parameter settings is also summarized.

Table S2

f_3-statistics of Hungarian and selected South Asian populations.

Pop A	Pop B	Pop C	F3 value	Std Err	Z score	SNP count
STU	HUN	PJL	-0.003761	0.000140	-26.782	76834
ITU	HUN	PJL	-0.003641	0.000141	-25.752	76845
BEB	HUN	PJL	-0.002865	0.000138	-20.743	80622

Negative F_3 values with significant Z score indicate complex relations (admixture) between the Hungarian and South Asian populations.

Figure S1

F_4(TSI, X; CHB; YRI) values of the analyzed datasets

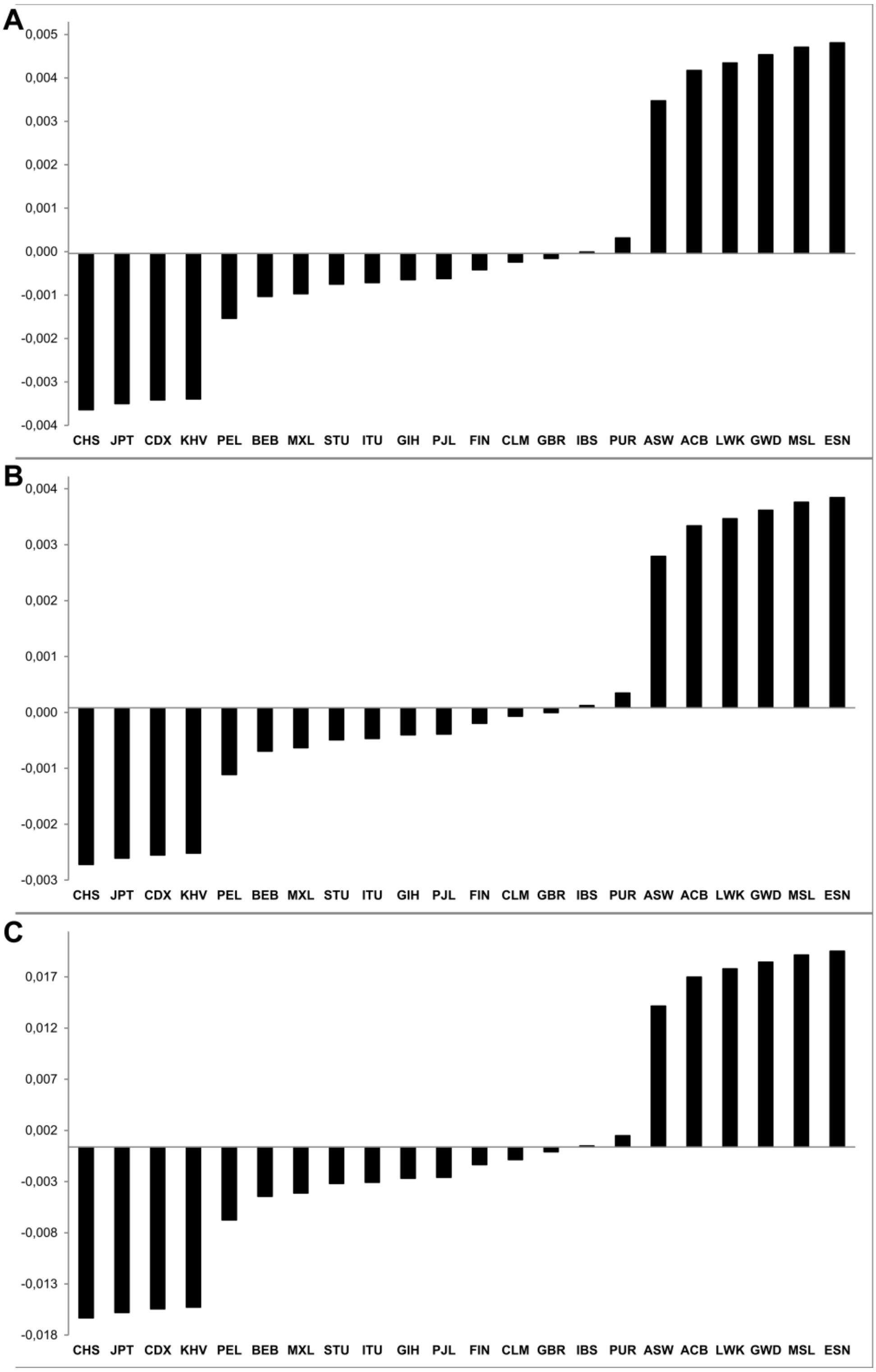

A) GENOME, B) EXOME and C) BEADCHIP, where X denotes the test population indicated on the x-axis.

Figure S2

Calculated Cross Validation error for different admixture models (K=3 to 10) for each dataset.

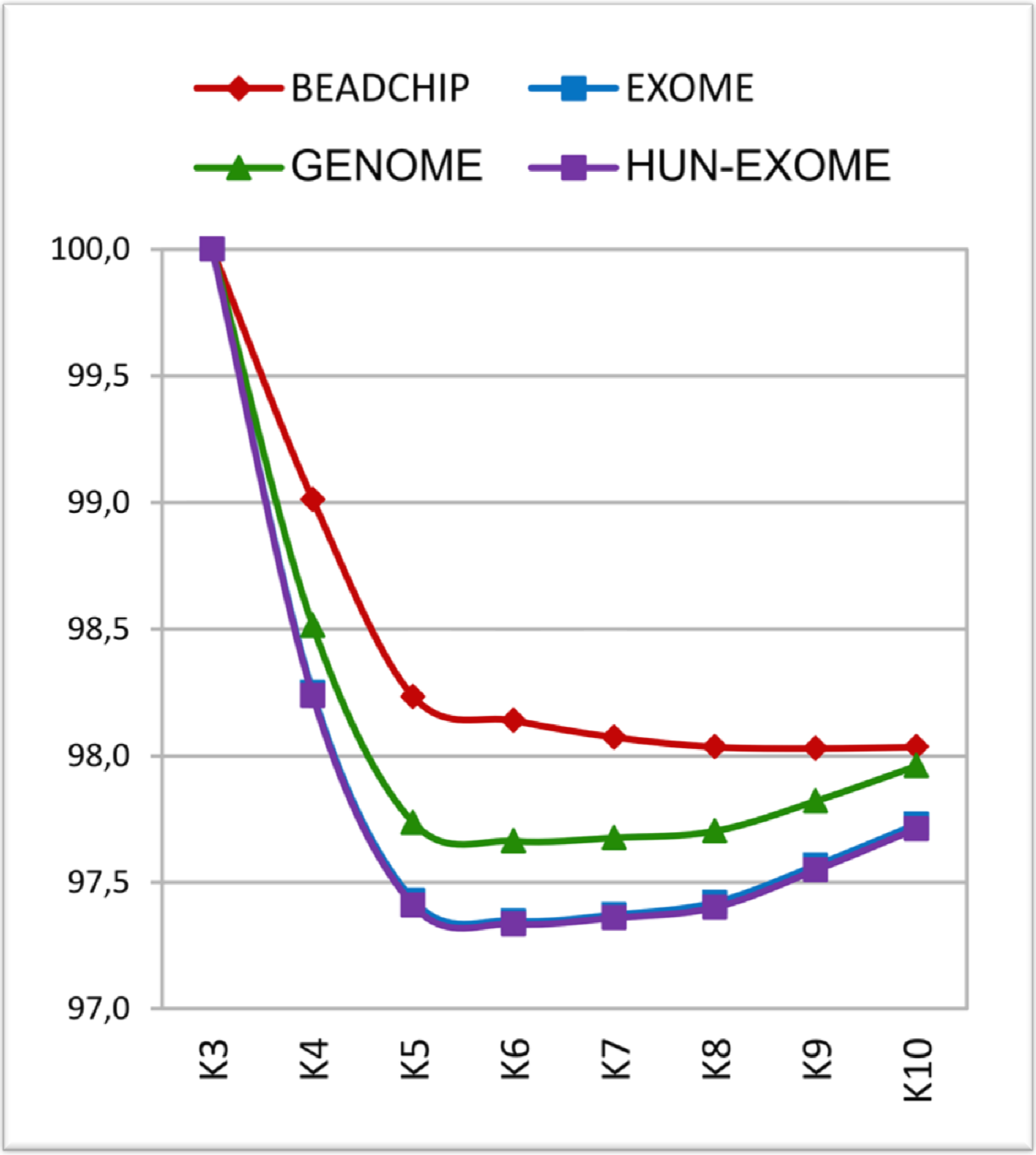

Figure S3

Admixture analysis of 25 populations using the GENOME, EXOME and BEADCHIP datasets with the K=6 admixture model.

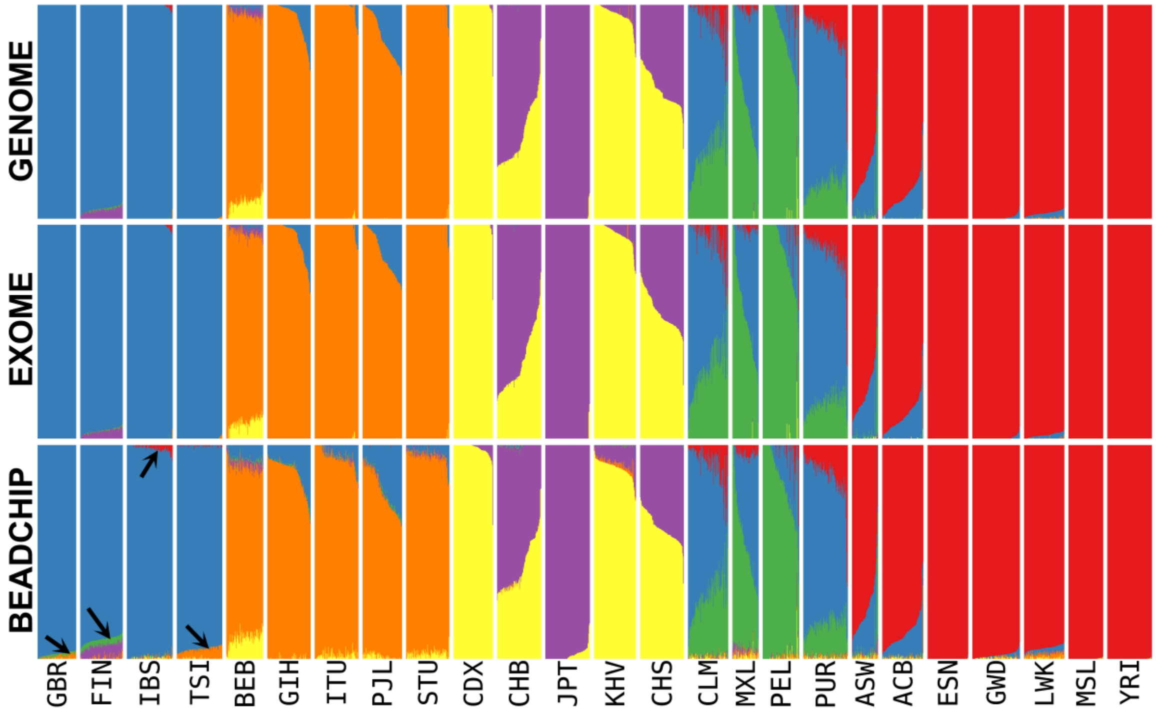

The arrows denote examples of minor genetic admixture components within the European super-population.

Figure S4

Admixture analysis of 25 populations using the GENOME, EXOME and BEADCHIP datasets with the K=9 admixture model.

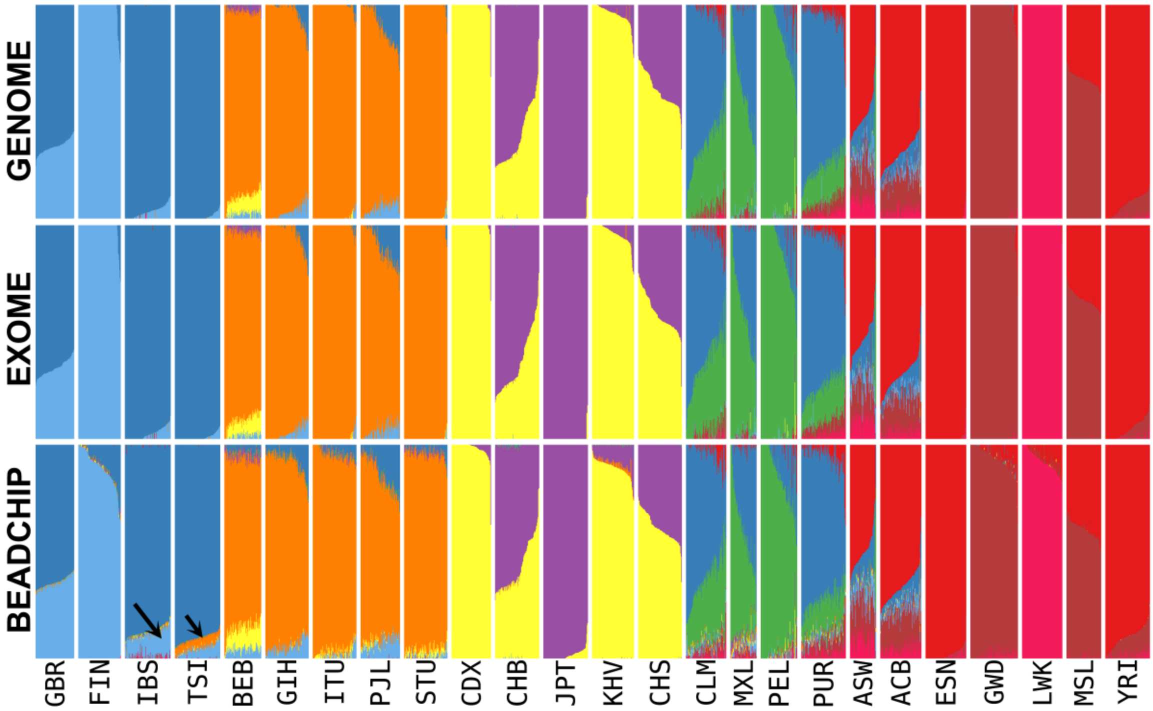

The arrows denote examples of minor genetic admix components within the European super-population.

Figure S5

PCA of the HUN-EXOME dataset

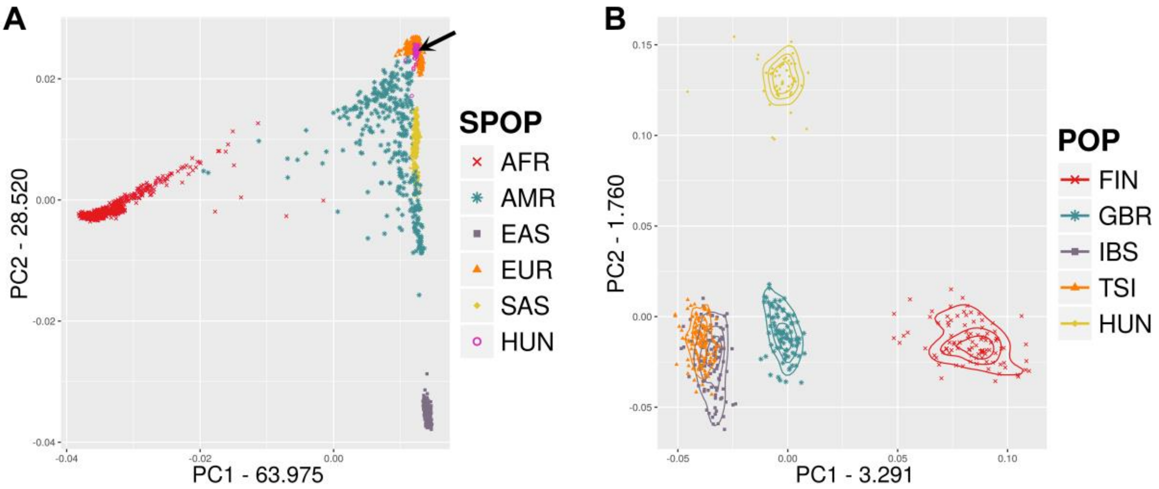

A) Hungarians (highlighted by the arrow) in relation to the super-population, **B)** Hungarians in relation to European populations.

Figure S6

F4(TSI, X; CHB, YRI) values of the HUN-EXOME dataset, where X denotes the test population indicated on the x-axis.

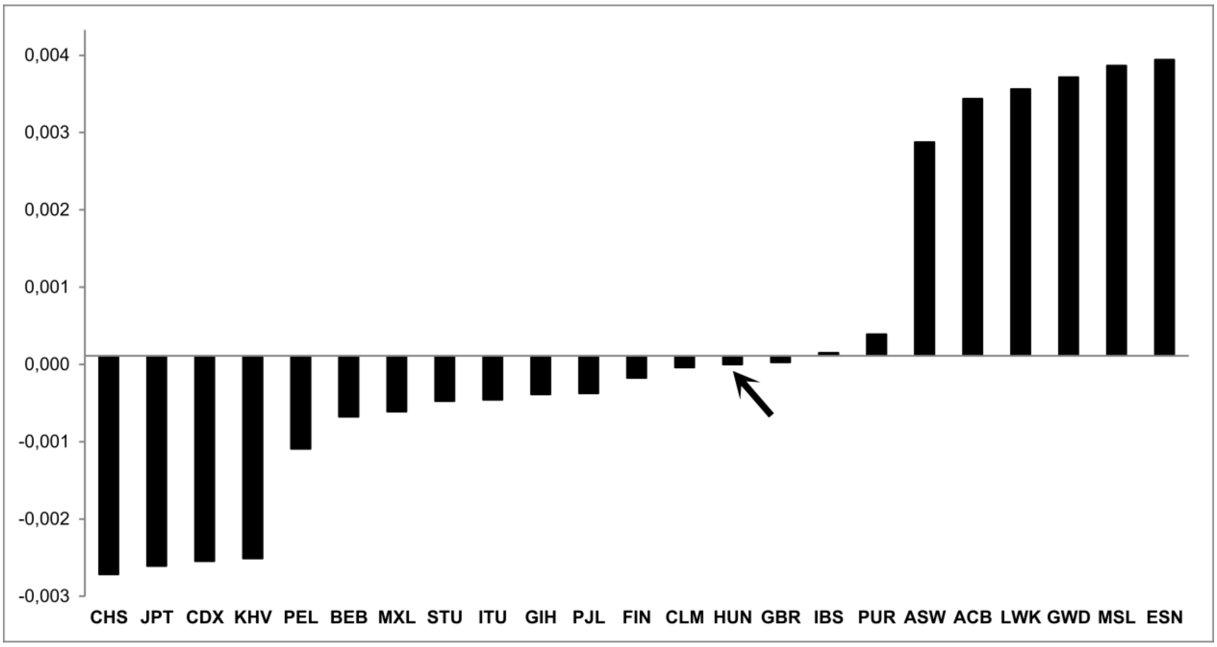

The arrow shows the relative position of Hungarians, indicating that they have higher East Asian admixture component than British but lower than Finnish population.

Figure S7

Admixture analysis of 25 populations using the HUN-EXOME dataset for the **A)** K=6 and **B)** K=9 admixture models.

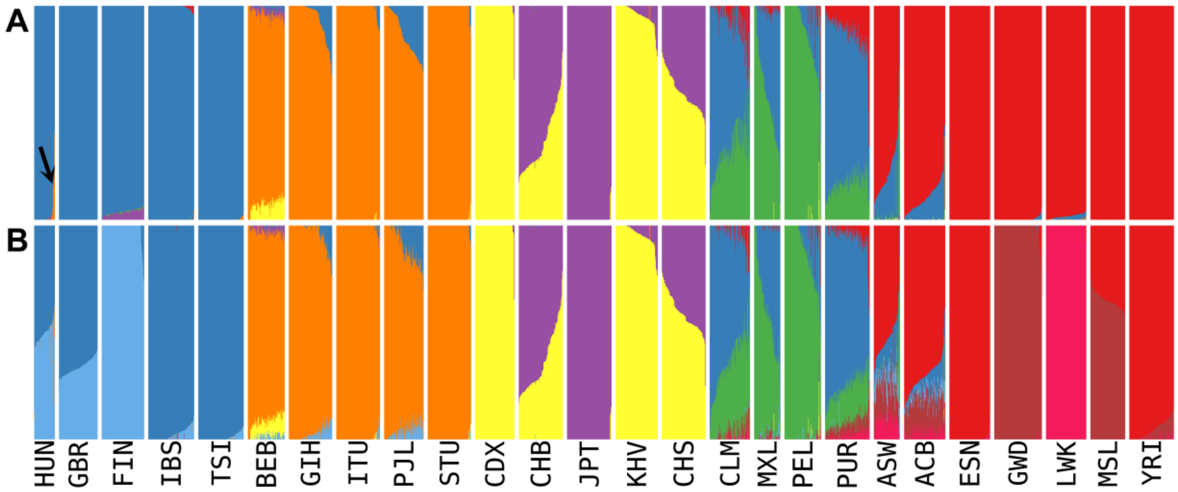

The arrow denotes Hungarian individuals with substantial South Asian admixture components.

Data

We have deposited the Supplementary Data to figshare:

https://figshare.com/s/e91794c7141a7eb16255
